# The Effects of Nonclinician Guidance on Effectiveness and Process Outcomes in Digital Mental Health Interventions: Systematic Review and Meta-analysis

**DOI:** 10.2196/36004

**Published:** 2022-06-15

**Authors:** Calista Leung, Julia Pei, Kristen Hudec, Farhud Shams, Richard Munthali, Daniel Vigo

**Affiliations:** 1 Department of Psychiatry Faculty of Medicine University of British Columbia Vancouver, BC Canada

**Keywords:** digital mental health, nonclinician guidance, e-Mental health intervention, internet-based intervention, mental health, task shifting, digital health, digital health intervention, patient outcome

## Abstract

**Background:**

Digital mental health interventions are increasingly prevalent in the current context of rapidly evolving technology, and research indicates that they yield effectiveness outcomes comparable to in-person treatment. Integrating professionals (ie, psychologists and physicians) into digital mental health interventions has become common, and the inclusion of guidance within programs can increase adherence to interventions. However, employing professionals to enhance mental health programs may undermine the scalability of digital interventions. Therefore, delegating guidance tasks to paraprofessionals (peer supporters, technicians, lay counsellors, or other nonclinicians) can help reduce costs and increase accessibility.

**Objective:**

This systematic review and meta-analysis evaluates the effectiveness, adherence, and other process outcomes of nonclinician-guided digital mental health interventions.

**Methods:**

Four databases (MEDLINE, Embase, CINAHL, and PsycINFO) were searched for randomized controlled trials published between 2010 and 2020 examining digital mental health interventions. Three journals that focus on digital intervention were hand searched; gray literature was searched using ProQuest and the Cochrane Central Register of Control Trials (CENTRAL). Two researchers independently assessed risk of bias using the Cochrane risk-of-bias tool version 2. Data were collected on effectiveness, adherence, and other process outcomes, and meta-analyses were conducted for effectiveness and adherence outcomes. Nonclinician-guided interventions were compared with treatment as usual, clinician-guided interventions, and unguided interventions.

**Results:**

Thirteen studies qualified for inclusion. Nonclinician-guided interventions yielded higher posttreatment effectiveness outcomes when compared to conditions involving control programs (eg, online psychoeducation and monitored attention control) or wait-list controls (*k*=7, Hedges *g*=–0.73; 95% CI –1.08 to –0.38). There were also significant differences between nonclinician-guided interventions and unguided interventions (*k*=6, Hedges *g*=–0.17; 95% CI –0.23 to –0.11). In addition, nonclinician-guided interventions did not differ in effectiveness from clinician-guided interventions (*k*=3, Hedges *g*=0.08; 95% CI –0.01 to 0.17). These results suggest that guided digital mental health interventions are helpful to improve mental health outcomes regardless of the qualifications of the individual performing the intervention, and that the presence of a nonclinician guide improves effectiveness outcomes compared to having no guide. Nonclinician-guided interventions did not yield significantly different adherence outcomes when compared with unguided interventions (*k*=3, odds ratio 1.58; 95% CI 0.51 to 4.92), although a general trend of improved adherence was observed within nonclinician-guided interventions.

**Conclusions:**

Integrating paraprofessionals and nonclinicians appears to improve the outcomes of digital mental health interventions, and may also enhance adherence outcomes (though this trend was nonsignificant). Further research should focus on the specific types of tasks these paraprofessionals can successfully provide (ie, psychosocial support, therapeutic alliance, and technical augmentation) and their associated outcomes.

**Trial Registration:**

PROSPERO International Prospective Register of Systematic Reviews CRD42020191226; https://www.crd.york.ac.uk/prospero/display_record.php?RecordID=191226

## Introduction

The 2017 World Psychiatric Association-Lancet Psychiatry Commission on the Future of Psychiatry highlighted digital psychiatry and the reform of traditionally structured mental health services as key priority areas for the future of the field [1]. Digital mental health interventions (or e-mental health interventions) have become increasingly prevalent in recent years, and research suggests that these interventions have similar effectiveness as in-person mental health treatment [2]. These interventions have been effective in addressing a range of mental health concerns and can reduce the severity of depression [3], anxiety, and stress, reduce eating disorder symptoms, improve social well-being [4], and reduce alcohol consumption [5].

In addition to generating positive health outcomes, offering mental health treatment through digital platforms offers several advantages over brick-and-mortar formats. A digital intervention’s inherent scalability enhances social welfare, protects patients from stigma and discrimination, and allows for low- and middle-income countries or geographically inaccessible areas to deploy critical mental health care that would otherwise be impractical due to insufficiencies in service infrastructure [6]. It can also be of use in higher-income countries, where it can provide increased convenience and accessibility for populations wishing to remain anonymous due to mental health stigma, reduce costs, broaden the reach of treatment, and increase the flexibility of treatment [7,8]. Digital interventions can also increase willingness to use mental health services: a study of US soldiers reported that 33% of those unwilling to utilize in-person counselling were willing to utilize a technology-based mental health treatment [9].

Digital mental health interventions have become widely available, but adherence has been poor [10]. Low adherence may subvert the effectiveness of digital mental health tools [11]. Implementing human support for digital interventions may offer a solution by improving adherence and effectiveness outcomes; this improvement may be mediated by the increased accountability that coaches provide through assistance, support, and scheduled contacts [12].

While human support is often provided by clinicians with positive effects [13,14], integrating professional clinicians into digital interventions can be costly and resource intensive. Engaging nonclinicians, such as lay workers and peers, offers a cost-effective way to address the gap in treatment [1]; shifting certain tasks that a professional would normally provide (such as developing a therapeutic alliance, providing weekly reminders for program completion, or general administrative tasks) onto a lesser-trained nonclinician coach can reduce costs and enable scaling up of digital interventions. The literature suggests this strategy can be effective; a meta-analysis of digital interventions for anxiety disorders did not identify significant differences in treatment outcomes between coaches of varying qualifications or levels of training [15]. Further, a systematic review of peer-to-peer interactions in digital interventions reported that peer support yielded positive effects on effectiveness and adherence outcomes alongside increased perceptions of social support for individuals with psychotic disorders [16]. Therefore, it seems intuitive to utilize paraprofessionals or peers to administer certain forms of support.

Despite the abundance of research on clinician-guided digital mental health interventions and studies suggesting the benefits of integrating nonclinicians, the pooled effects of nonclinician-guided digital interventions on a broader range of mental health and substance use issues do not appear to have been formally evaluated. As such, we conducted a systematic literature review and meta-analysis examining the effectiveness, adherence, and other process outcomes of nonclinician-guided digital mental health interventions compared to clinician-guided and unguided digital mental health interventions and to treatment as usual.

## Methods

### Inclusion and Exclusion Criteria

Randomized controlled trials (RCTs) qualified for inclusion if (1) they evaluated a digital intervention addressing clinical or subthreshold mental health, substance use–related issues, or direct determinants of these issues; (2) the digital intervention targeted the mental health of the individual receiving the intervention (eg, parenting interventions targeting the mental health of the child were excluded); (3) the digital intervention targeted primary mental health outcomes (as opposed to mental health outcomes secondary to physical conditions); (4) the digital intervention was supported by a nonclinician (eg, a peer, research assistant, or other layperson); (5) the control groups were (a) offered an unguided intervention, (b) offered clinician-guided intervention (ie, by a psychiatrist, psychologist, therapist, social worker, graduate student in a mental health–related field, or student completing clinical practicum training), (c) offered an in-person intervention, (d) put on a wait-list for a digital intervention or offered any form of “treatment as usual,” or (e) offered an active control intervention (eg, monitored attention control or informative emails); (6) they included subjects between 16 and 64 years old; and (7) they reported effectiveness, adherence, or other process outcomes as primary outcomes. The inclusion criteria were piloted on small samples of studies and refined accordingly. Any disagreements were resolved through discussion or consultation with a third researcher (DV). Only English-language or English-translated publications were included.

### Search Strategy

A systematic search of literature published between 2010 and 2020 was conducted in July 2020. The publication time frame was selected to ensure included technologies were current rather than outdated (eg, video conferencing vs CD-ROM); thus, the findings are applicable to the current landscape of digital intervention research. Four databases (MEDLINE, Embase, CINAHL and PsycINFO) were searched using MeSH terms, keywords, and text (Multimedia Appendix 1).

Three theme-specific journals (Internet Interventions, Lancet Digital Health and the Journal of Medical Internet Research) were also hand searched. ProQuest and the Cochrane Central Register of Controlled Trials (CENTRAL) were searched for gray literature. Forward and backward reference chaining of included studies was performed and relevant reviews found through screening were searched for pertinent papers. Emails were sent to authors of relevant protocols and conference proceedings to ascertain whether an RCT had been conducted. The review protocol was registered on the International Prospective Register of Systematic Reviews (PROSPERO) before data extraction was initiated (CRD42020191226).

### Study Selection

Titles and abstracts were independently screened by 2 researchers (CL and JP), then full text reports were independently evaluated by the same 2 researchers. Conflicts were resolved through discussion or, when needed, consultation with a third researcher (DV). Covidence, a web-based screening tool (Veritas Health Innovation), was used to facilitate collaborative screening [17].

**Figure 1:**
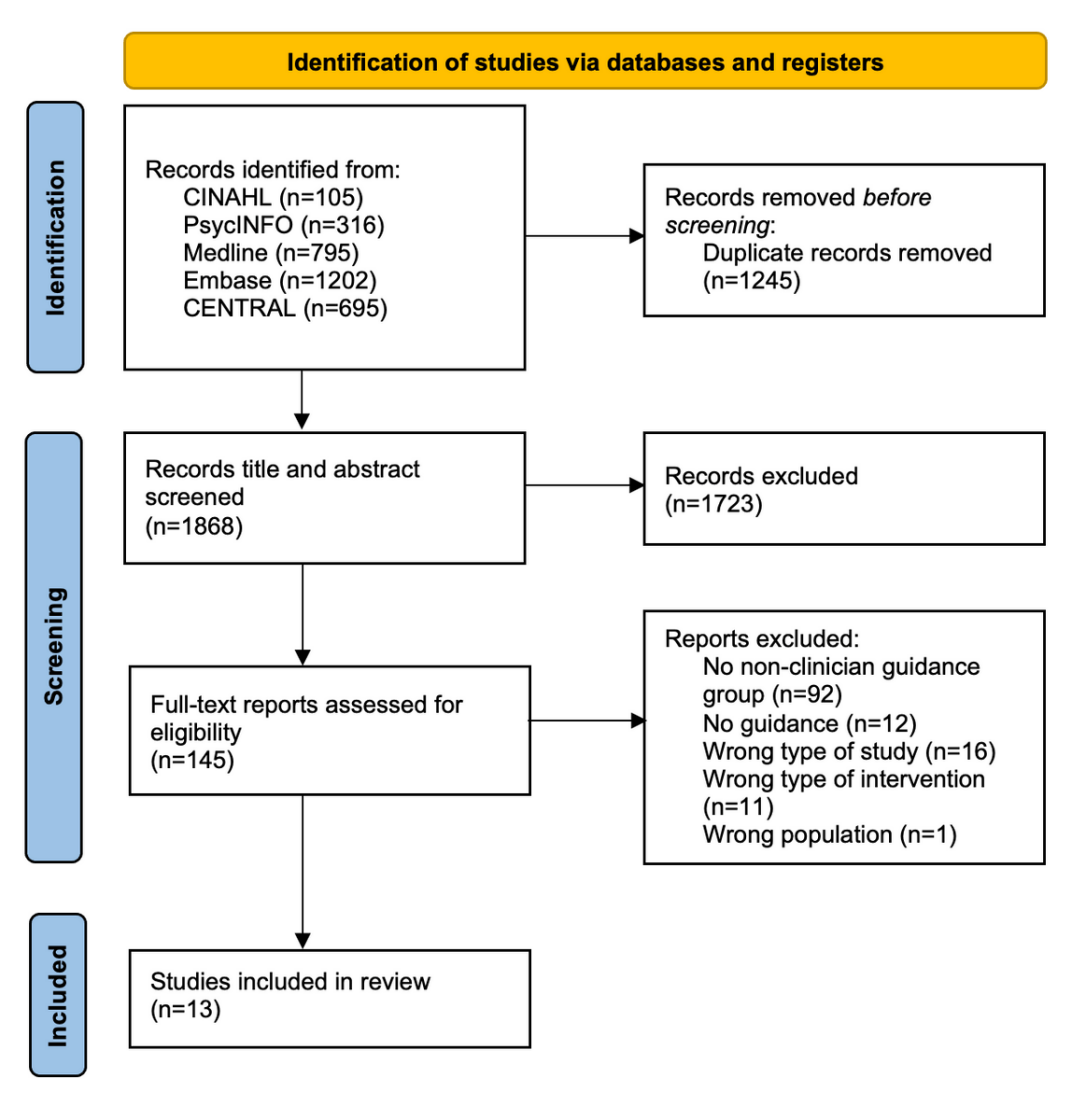
PRISMA (Preferred Reporting Items for Systematic Reviews and Meta-Analyses) flowchart.

Through searching, 3113 studies were identified. After deduplication, titles and abstracts of 1868 studies and full texts of 145 studies were screened. Thirteen studies qualified for inclusion. The PRISMA (Preferred Reporting Items for Systematic Reviews and Meta-Analyses) flow diagram is presented in Figure 1.

### Data Extraction and Study Characteristics

CL extracted data from the included articles (N=13), and JP validated the extracted data. Disagreements were resolved through discussion. Extracted data included intervention name, location, duration, design, arms, sample size, targeted mental health problem or disorder, theoretical model, nonclinician guide qualification, effectiveness outcomes, adherence outcomes, process outcomes, and results for these outcomes (results are shown in Table 1; additional information is shown in Multimedia Appendix 2). There were 3227 participants across the 13 studies. Treatment durations ranged from 4 to 12 weeks and sample sizes ranged from 30 to 1405 participants. The majority of interventions targeted mood and anxiety disorders (n=7). Other studies targeted well-being (n=1), stress (n=1), posttraumatic stress disorder (n=1), obsessive compulsive disorder (n=1), bipolar disorder (n=1), and substance use (n=2). Cognitive behavioral therapy was the most common theoretical model underpinning the interventions.

**Table 1:** Summary of results.

Study, year			Results
**An et al, 2013 [18]**			
	Targeted disorder	Substance use (smoking)	
	Subjects for each study condition, n	Nonclinician, 456; unguided, 473; control; 476	
	Effectiveness outcome (30-day smoking abstinence), %	Nonclinician, 14%; unguided, 11%; control, 9%	
**Arjadi et al, 2018 [19]**			
	Targeted disorder	Depression	
	Subjects for each study condition, n	Nonclinician, 159; control, 154	
	Effectiveness outcome (Patient Health Questionnaire-9 score), mean (SD)	Nonclinician, 8.5 (5.74); control, 10.83 (6.21)	
**Day et al, 2013 [20]**			
	Targeted disorder	Depression	
	Subjects for each study condition, n	Nonclinician, 33; control (delayed access), 33	
	Effectiveness outcome (Depression, Anxiety and Stress Scale depression score), mean (SD)	Nonclinician, 10.43 (4.49); control, 14.6 (9.51)	
	Adherence outcome (completion of all modules), %	Nonclinician, 61%; control, N/A^a^ (adherence outcomes unreported)	
	Process outcome	Usefulness	
	Results	The average usefulness rating of the overall modules was 6.78/10 (ranging from 1, “not useful at all,” to 10, “extremely useful”).	
**Dirkse et al, 2020 [21]**			
	Targeted disorder	Depression	
	Subjects for each study condition, n	Nonclinician, 41; unguided, 42	
	Effectiveness outcome (Patient Health Questionnaire-9 score), mean (SD)	Nonclinician, 4.83 (2.7); unguided, 5.51 (4.5)	
	Adherence outcome (completion of all modules), %	Nonclinician, 93%; unguided, 81%	
	Process outcome	Satisfaction	
	Results	A total of 85% of unguided and 90% of nonclinician-guided participants were either “satisfied” or “very satisfied” with the course (no significant difference), 93% of unguided and 100% of nonclinician-guided participants were either “satisfied” or “very satisfied” with the quality of the lessons and the materials (no significant difference); nonclinician-guided participants had significantly higher levels of satisfaction with the level of support, though both groups had relatively high satisfaction (96% of participants overall were “satisfied” or “very satisfied”).	
**Farrer et al, 2011 [22]**			
	Targeted disorder	Depression	
	Subjects for each study condition, n	Nonclinician, 41; unguided, 38; control, 35	
	Effectiveness outcome (Center for Epidemiologic Studies Depression Scale score), mean (SD)	Nonclinician, 21 (12.4); unguided, 24.4 (13.6); control, 35.1 (13.9)	
	Adherence outcome (minimum dose: 3/5 modules), %	Nonclinician, 37.7%; unguided, 31.6%; control, N/A (received no intervention)	
	Adherence outcome (completion of all modules), %	Nonclinician, 17.8%; unguided, 15.8%; control, N/A (received no intervention)	
**Flynn et al, 2020 [23]**			
	Targeted disorder	Mental well-being	
	Subjects for each study condition, n	Nonclinician, 30; unguided, 30	
	Effectiveness outcome (Warwick-Edinburgh Mental Wellbeing Scale score), mean (SD)	Nonclinician, 48.43 (12.66); unguided, 42.88 (9.66)	
	Adherence outcome (completion of all modules), %	Nonclinician, 52%; unguided, 43%	
**Heber et al, 2016 [24]**			
	Targeted disorder	Stress	
	Subjects for each study condition, n	Nonclinician, 132; control (delayed access), 132	
	Effectiveness outcome (Perceived Stress Scale-10 score), mean (SD)	Nonclinician, 17.88 (6.17); control, 22.96 (6.07)	
	Adherence outcome (completion of all modules), %	Nonclinician, 70.5%; control, N/A (adherence outcomes unreported)	
	Process outcome	Satisfaction	
	Results	A total of 92.2% of participants were “satisfied in an overall, general sense” (ie, either “very satisfied” or “mostly satisfied”).	
**Kobak et al, 2015 [25]**			
	Targeted disorder	Obsessive compulsive disorder	
	Subjects for each study condition, n	Clinician, 31; nonclinician, 28; unguided, 28	
	Effectiveness outcome (Yale Brown Obsessive Compulsive Scale score), mean (SD)	Clinician, 15.32 (7.04); nonclinician, 15.61 (5.88); unguided, 16.32 (6.97)	
	Process outcomes	Satisfaction, usability	
	Results	A total of 98% of participants “agreed” or “strongly agreed” with the statement that “they were satisfied with bt steps.” For usability, the mean total system usability score was 83.5/100 (between “good” and “excellent”).	
**Possemato et al, 2019^b^** **[26]**			
	Targeted disorder	Posttraumatic stress disorder and hazardous drinking	
	Subjects for each study condition, n	Nonclinician, 15; unguided, 15	
	Effectiveness outcome (Posttraumatic Stress Disorder Checklist—Military score), mean (SD)	Nonclinician, 41.78 (14.90); unguided, 43.16 (13.42)	
	Process outcome	Satisfaction	
	Results	A total of 78% of participants were “very satisfied.”	
**Proudfoot et al, 2012 [27]**			
	Targeted disorder	Bipolar disorder (perception of illness)	
	Subjects for each study condition, n	Nonclinician, 139; unguided, 141; control, 139	
	Adherence outcome (minimum dose; 4/8 module workbooks)	Nonclinician, 79.9%; unguided, 69.1%; control, N/A (received no intervention)	
	Adherence outcome (completion of all modules)	38.8% across 3 groups	
**Robinson et al, 2010 [28]**			
	Targeted disorder	Generalized anxiety disorder	
	Subjects for each study condition, n	Clinician, 47; nonclinician, 50; control (delayed access), 48	
	Effectiveness outcome (General Anxiety Disorder-7 score), mean (SD)	Clinician, 5.55 (4.73); nonclinician, 6.02 (3.43); control, 11.25 (4.70)	
	Adherence outcome (completion of all modules), %	Clinician, 74%, nonclinician, 80%; control, N/A (received no intervention)	
	Process outcome	Satisfaction	
	Results	A total of 87% of participants in the nonclinician-guided and clinician-guided groups were either “very satisfied” or “mostly satisfied” with the overall program (no significant difference).	
**Rosso et al, 2017 [29]**			
	Targeted disorder	Depression	
	Subjects for each study condition, n	Nonclinician, 37; control, 40	
	Effectiveness outcome (Hamilton Depression Rating Scale-17 score), mean (SD)	Nonclinician, 9.17 (6.92), control, 14.05 (5.34)	
	Adherence outcome (completion of all modules), %	Nonclinician, 92%; control, 75%	
**Titov et al, 2010 [30]**			
	Targeted disorder	Depression	
	Subjects for each study condition, n	Clinician, 46; nonclinician, 41; control, 40	
	Effectiveness outcome (Beck Depression Inventory-II score), mean (SD)	Clinician, 14.59 (11.12); nonclinician, 15.29 (9.81); control, 26.15 (10.14)	
	Adherence outcome (completion of all modules), %	Clinician, 80%; nonclinician, 80%; control, N/A (adherence outcomes unreported)	
	Process outcome	Satisfaction	
	Results	A total of 87% of participants in the nonclinician-guided or clinician-guided groups were either “very satisfied” or “mostly satisfied” with the overall program (no significant difference).	

^a^N/A: not applicable.

^b^Possemato reported a nonclinician intervention retention rate of 93% and unguided intervention retention rate of 73% but did not define “intervention retention.”

### Quality Assessment

Two researchers (CL and JP) independently assessed risk of bias using Version 2 of the Cochrane risk-of-bias tool version 2 (RoB 2) [31]. Disagreements were resolved through discussion with a third researcher (DV). The RoB 2 evaluates the risk of bias associated with randomization, deviation from the intended intervention, missing outcome data, outcome measurement, and selection of the reported result. Each domain was assigned a judgment of “low risk of bias,” “some concerns,” or “high risk of bias.”

### Outcomes

The effectiveness outcomes described changes in mental health symptomology or substance use behaviors. Five studies included primary effectiveness outcomes, so 2 mental health clinicians were consulted in developing a hierarchy of outcomes [32]. When multiple mental health concerns were fully assessed as primary outcomes, the clinical metric that was reported as a primary outcome (eg, obsessive compulsive disorder over stress) among a greater number of studies was selected. When multiple instruments were used, the clinical outcomes were prioritized and reported (eg, Posttraumatic Stress Disorder Checklist-Military, which assesses posttraumatic stress disorder, was selected over the World Health Organization Quality of Life Questionnaire, which assesses quality of life). When multiple clinical instruments were reported, the most thorough instrument was reported (eg, Beck Depression Inventory, a 21-item inventory, was selected over the Patient Health Questionnaire, a 9-item inventory).

Adherence outcomes were defined as the proportion of participants that either fully completed the intervention or completed a defined minimum dose of the intervention; both full completion and minimum dose completion outcomes were included in the adherence meta-analysis due to the small number of studies reporting minimum dose adherence. Process outcomes consisted of participant satisfaction, intervention usefulness, and digital tool usability.

### Data Analysis

A random effects model was used to conduct all meta-analyses [33]. This model assumes a distribution of true effect sizes, accounting for the different populations that each publication studied [32]. Outcomes were analyzed using the meta [34], metafor [35], and esc [36] packages in RStudio (version 3.6.2; R Foundation) (Multimedia Appendix 3 includes the full code).

Three meta-analyses of effectiveness outcomes were conducted: nonclinician-guided interventions versus clinician-guided interventions [25,28,30], nonclinician-guided interventions versus unguided interventions [18,21-23,25,26], and nonclinician-guided interventions versus controls (ie, wait-list or monitored attention control) [18-20,22,24,28-30]. Unguided interventions provided the same content as clinician or nonclinician-guided interventions (without the guide component), whereas control programs may not have included an intervention (ie, they used a wait-list) or may have provided different content than was utilized in the intervention arm. This meta-analytic approach avoids conflating active treatment arms with wait-list controls and more clearly elucidates the effects of nonclinician guidance. Meta-analyses of posttreatment effects were conducted for all 3 comparisons, and meta-analyses of follow-up effects were conducted for the nonclinician-guided intervention versus unguided intervention and nonclinician-guided intervention versus control comparisons. Although both posttreatment standardized mean difference (SMD) and pretest-posttest control group (*d*_ppc2_) [37] effect sizes have been utilized in similar meta-analyses [38,39], we determined that the posttreatment SMD effect size was most appropriate due to the lack of pre-post correlation values available from the included studies and the criticisms of pre-post effect size methods [40]. A sensitivity analysis was conducted comparing the 2 methods and resulted in the same pattern of findings. Hedges *g* effect sizes were used alongside their respective 95% CIs to correct for small sample sizes [41] and were interpreted according to recommendations [42] (small effect: <0.20; medium effect: 0.21-0.50; and large effect: 0.51-0.80). When a high level of heterogeneity was observed in the meta-analysis of nonclinician-guided interventions and controls, a meta-regression evaluating the effects of the control group type (wait-list vs control intervention) was conducted to determine whether these effects contributed to the heterogeneity.

Most studies reported reductions in symptoms as negative effects. A minority of papers reported effects that increased with symptom reduction, so these outcomes were reverse coded [18,23]. Proudfoot et al (2012) was excluded from the meta-analyses because only coefficients (rather than group scores) were reported [27], and the authors could not be reached to obtain the necessary data.

One meta-analysis was conducted for adherence outcomes, comparing nonclinician-guided interventions and unguided interventions, as there was insufficient data to conduct additional comparisons. Odds ratios were used as effect sizes [43]. Study selection for this meta-analysis was based on whether the results of the publication described full intervention completion rates in nonclinician-guided groups and unguided groups (3 publications satisfied these criteria; Table 1). No meta-analysis was conducted for other process outcomes (ie, satisfaction, usability, and usefulness) due to the small number of studies reporting these outcomes, but findings have been summarized below.

## Results

### Quality Assessment

RoB 2 was used to conduct an assessment of the methodological quality of the 13 included papers (Table 2). Separate assessments were conducted for effectiveness, adherence, and process outcomes (Multimedia Appendix 4).

Effectiveness outcomes were assessed for 12 papers: 4 studies scored “high risk,” 5 studies scored “some concerns,” and 4 studies scored “low risk.” High risk was most commonly driven by domain 3 (missing outcome data) and domain 4 (measurement of the outcome). High risk of bias was associated with domain 3 when experimenters did not adequately correct for bias stemming from missing data or did not describe doing so, since participants who completed follow-up measures were more likely to have more favorable efficacy outcomes than dropouts.

Adherence outcomes were assessed for the 12 papers that reported adherence outcomes. Five types of adherence outcomes were evaluated: completion of the whole intervention, completion of a minimum dose, percentage completion of each intervention module, mean number of modules completed, and intervention retention. Papers that reported multiple adherence outcomes were assessed for each adherence outcome reported, though each of these papers scored the same overall risk of bias for each adherence outcome. Two studies scored “high risk,” 7 studies scored “some concerns,” and 3 studies scored “low risk.” High risk was driven by domain 2 (deviations from intended interventions) and domain 5 (selection of the reported result).

Process outcomes (satisfaction, usefulness, and usability) were assessed for 7 papers; 6 studies scored “high risk” and 1 study scored “some concerns.” High risk was most associated with domain 3 (missing outcome data); since many of these outcomes were subjective in nature, participants who completed follow-up measures may have been more likely to rate these outcomes favorably than dropouts.

**Table 2:** Cochrane risk-of-bias tool version 2 summary.

Study, year			Randomization process		Deviations from intended interventions		Missing outcome data		Measurement of the outcome		Selection of the reported result		Overall
**Effectiveness outcome assessments**													
	An et al, 2013 [18]	Low		Some concerns		High		Some concerns		High		High	
	Arjadi et al, 2018 [19]	Low		Low		Low		Low		Low		Low	
	Day et al, 2013 [20]	Low		Low		Low		Low		Some concerns		Some concerns	
	Dirkse et al, 2020 [21]	Low		Low		Low		Low		Some concerns		Some concerns	
	Farrer et al, 2011 [22]	Some concerns		Low		Low		Low		High		High	
	Flynn et al, 2020 [23]	Low		High		High		Low		Some concerns		High	
	Heber et al, 2016 [24]	Low		Low		Low		Low		Some concerns		Some concerns	
	Kobak et al, 2015 [25]	Some concerns		High		Low		Low		Some concerns		High	
	Possemato et al, 2019 [26]	Low		Low		Low		Some concerns		Some concerns		Some concerns	
	Robinson et al, 2010 [28]	Some concerns		Low		Low		Some concerns		Low		Some concerns	
	Rosso et al, 2017 [29]	Low		Low		Low		Low		Some concerns		Some concerns	
	Titov et al, 2010 [30]	Some concerns		Low		High		Low		Some concerns		High	
**Adherence outcome assessments—completion of whole intervention**													
	Day et al, 2013 [20]	Low		Low		Low		Low		Low		Low	
	Dirkse et al, 2020 [21]	Some concerns		Low		Low		Low		Low		Some concerns	
	Farrer et al, 2011 [22]	Some concerns		Low		Low		Low		Low		Some concerns	
	Flynn et al, 2020 [23]	Low		High		Low		Low		Low		High	
	Heber et al, 2016 [24]	Low		Some concerns		Low		Low		Low		Some Concerns	
	Proudfoot et al, 2012 [27]	Low		Low		Low		Low		High		High	
	Robinson et al, 2010 [28]	Some concerns		Low		Low		Low		Low		Some concerns	
	Rosso et al, 2017 [29]	Low		Low		Low		Low		Low		Low	
	Titov et al, 2010 [30]	Some concerns		Low		Low		Low		Low		Some concerns	
**Adherence outcome assessments—completion of minimum dose**													
	An et al, 2013 [18]	Low		Some concerns		Low		Low		Low		Some concerns	
	Farrer et al, 2011 [22]	Some concerns		Low		Low		Low		Low		Some concerns	
	Proudfoot et al, 2012 [27]	Low		Low		Low		Low		High		High	
**Adherence outcome assessments—percentage completion of each intervention module**													
	Arjadi et al, 2018 [19]	Low		Low		Low		Low		Low		Low	
	Farrer et al, 2011 [22]	Some concerns		Low		Low		Low		Low		Some concerns	
**Adherence outcome assessments—mean number of modules completed**													
	Possemato et al, 2019 [26]	Low		Low		Low		Low		Some concerns		Some concerns	
**Process outcome assessments**													
	Day et al, 2013 [20]	Low		Low		High		High		Low		High	
	Dirkse et al, 2020 [21]	Low		Low		Low		High		Some concerns		High	
	Heber et al, 2016 [24]	Low		Some concerns		High		Low		Low		High	
	Kobak et al, 2015 [25]	Some concerns		High		Low		Some concerns		Some concerns		High	
	Possemato et al, 2019 [26]	Low		Low		High		High		Low		High	
	Robinson et al, 2010 [28]	Some concerns		Low		High		Low		Low		High	
	Titov et al, 2010 [30]	Some concerns		Low		High		High		Some concerns		High	

### Primary Posttreatment Effectiveness Outcomes

#### Nonclinician Versus Clinician

**Figure 2:**
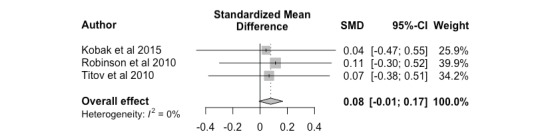
Nonclinician versus clinician, posttreatment. SMD: standardized mean difference.

The overall effect size from 3 studies was 0.08 (95% CI –0.01 to 0.17), indicating nonclinician-guided interventions did not significantly differ from clinician-guided interventions with respect to participant mental health outcomes. The distribution of effect sizes was homogeneous *(P=*.98) and is shown in Figure 2 as a forest plot.

#### Nonclinician Versus Unguided

**Figure 3:**
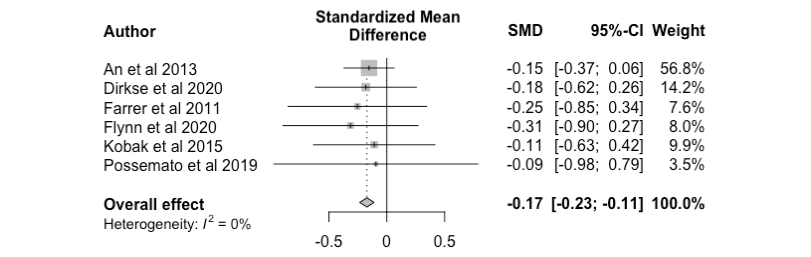
Nonclinician versus unguided, posttreatment. SMD: standardized mean difference.

The overall effect size (*k*=6, Hedges *g*=-0.17; 95% CI –0.23 to –0.11) between nonclinician-guided interventions and unguided interventions was significant. This small effect size indicates that digital mental health interventions were more effective when paraprofessionals or nonclinicians were involved in the intervention. The distribution of effect sizes was homogeneous *(P=*.99), ranging from –0.31 to –0.09, and is shown in Figure 3 as a forest plot.

#### Nonclinician Versus Control

**Figure 4:**
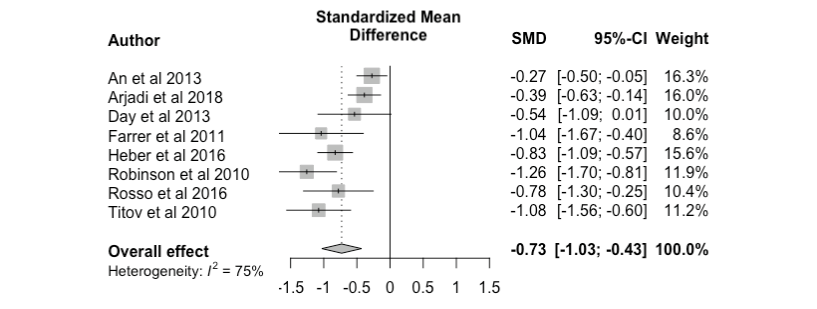
Nonclinician versus control, posttreatment. SMD: standardized mean difference.

Based on 8 studies, the overall effect size was –0.73 (95% CI –1.08 to –0.38). This significant, large effect size indicates nonclinician-guided interventions yielded higher posttreatment effectiveness outcomes than control programs (eg, online psychoeducation and monitored attention control) or wait-list controls. The distribution of effect sizes was heterogeneous *(P<*.001), ranging from –1.26 to –0.27, and is shown in Figure 4 as a forest plot. The heterogeneity was further examined through a meta-regression using type of control (wait-list control, *k*=5 [20,22,24,28,30] or control intervention program, *k*=3 [18,19,29]). Results from the meta-regression indicate that variability in the observed effect sizes can be explained by whether the study implemented a wait-list control or control intervention program (*k*=8, *R*^2^=94.32%, *P*=.23).

### Follow-up Outcomes

#### Nonclinician Versus Unguided

**Figure 5:**
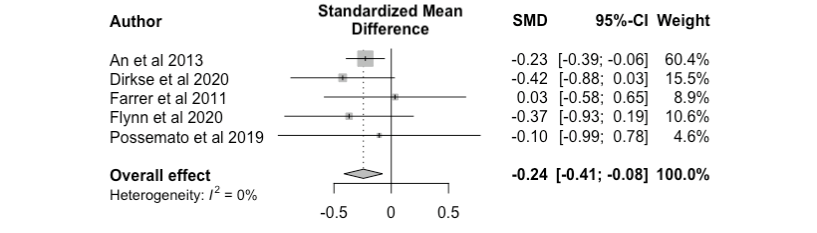
Nonclinician versus unguided, follow up. SMD: standardized mean difference.

Nonclinician-guided interventions yielded higher effectiveness outcomes than unguided interventions at follow-up, with a medium effect size (*k*=5, Hedges *g*=-0.24; 95% CI –0.41 to –0.08). The distribution of effect sizes was homogeneous (*P=*.79); results are shown in Figure 5 as a forest plot.

#### Nonclinician Versus Control

**Figure 6:**
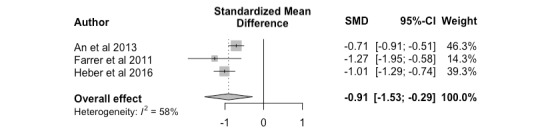
Nonclinician versus control, follow up.

Overall, nonclinician-guided interventions exhibited sustained improvement in effectiveness outcomes when compared to conditions involving wait-list controls and monitored attention controls at follow-up assessments; a large effect size was obtained (*k*=3, Hedges *g*=-0.91; 95% CI –1.53 to –0.29). Results are shown in Figure 6 as a forest plot. As a high level of heterogeneity was obtained, a meta-regression was conducted as a sensitivity analysis. The dependent variable was the effect size obtained from each study, and the explanatory variable was the type of control (wait-list control or control intervention program). The results indicated that all heterogeneity was accounted for by the type of control (*k*=3, *R*^2^=100%, *P*=.50), but this result could have been influenced by the minimal number of studies.

### Adherence Outcomes

Of the 13 studies, 9 reported the percentage of participants who completed the intervention [18,20,21,23,24,27-30]; 2 studies reported the percentage of participants who completed a defined minimum dose [22,27]; 2 studies reported the percentage of participants who completed each module [21,24], and 1 study reported the “intervention retention” percentage [26]. There was wide variation in adherence rates between the studies for both minimum dose and full completion measures. Minimum dose completion rates ranged from 31.6% to 79.9% and intervention completion rates ranged from 15.8% to 93%.

**Figure 7:**
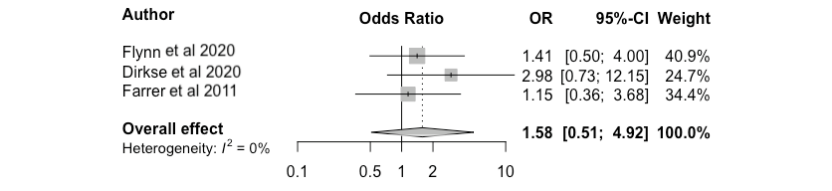
Adherence outcomes. OR: odds ratio.

Full completion adherence rates in nonclinician-guided and unguided groups were compared in 3 studies [21-23] and were therefore pooled in a meta-analysis; all 3 studies reported higher adherence rates in the nonclinician-guided groups. The meta-analysis indicated no significant effects on adherence outcomes in nonclinician versus unguided interventions (*k*=3, odds ratio 1.58 (95% CI 0.51 to 4.92)), although there was a general trend toward improved adherence outcomes when a nonclinician was involved (Figure 7).

Of the 2 studies that compared adherence rates in nonclinician-guided and clinician-guided groups, Robinson et al [28] reported higher adherence rates in the nonclinician-guided group, and Titov et al [30] reported the same adherence rates in each group. One study compared adherence rates in nonclinician-guided and monitored-attention control groups and reported higher adherence rates in the nonclinician-guided group [29]. Only 1 study reported the significance of the between-group difference and found that the nonclinician-guided group had significantly higher rates of intervention completion than the unguided group [28].

### Process Outcomes: Satisfaction, Usefulness, and Usability Results

Of the 13 studies, 6 evaluated participant satisfaction, 1 measured usefulness ratings of modules, and 1 measured system usability (Table 1). To measure participant satisfaction, 2 studies used questionnaires based on the Credibility/Expectancy Questionnaire [28,30], 2 studies used the Client Satisfaction Questionnaire [24,26], and 2 studies appeared to generate their own satisfaction measures [21,25]. The usefulness rating did not appear to be based on a preexisting scale [20]; system usability was measured using the System Usability Scale [25].

All studies reported that at least 78% of participants were satisfied with the intervention. Three studies compared satisfaction between groups. Two studies [28,30] found no significant difference in satisfaction between nonclinician-guided and clinician-guided groups. Dirkse et al [21] found no significant difference in intervention satisfaction between unguided and nonclinician-guided groups but reported that nonclinician-guided participants had significantly higher levels of satisfaction with the level of support. Day et al [20] reported a mean usefulness rating of 6.78/10 across guided and unguided groups. Kobak et al [25] reported a mean total system usability score of 83.5/100 across guided and unguided groups, which was between “good” and “excellent.”

## Discussion

### Main Results

#### Guided Versus Unguided and Control Interventions

Our meta-analysis indicates that guided digital mental health interventions significantly improve effectiveness outcomes compared to both control (intervention programs and wait-list) and unguided interventions. These results align with a seminal systematic review of guided digital mental health interventions by Baumeister et al, which reported that guided interventions were more favorable than unguided interventions [13]. Two previous meta-analyses also concluded that significant improvements in effectiveness were associated with guide involvement [44,45]. It is interesting and noteworthy that our results align with these previous meta-analyses [13,44,45], as these studies examined digital mental health intervention research published from 2002 to 2013—a period of time when the technological landscape was vastly different from today. Collectively, these findings suggest that the beneficial effects of guidance in digital mental health interventions have been sustained through large shifts in both use of and attention to technology and come at a time when digital mental health interventions are critical to meet increasing need [14,46]. As additional digital interventions are designed and deployed, administrators, developers, and user groups (such as patients) must be aware of the potential contributions of guides and consider these benefits when attempting to optimize mental health intervention outcomes.

#### Nonclinician- Versus Clinician-Guided Interventions

Nonclinician-guided interventions were associated with greater effectiveness compared to unguided interventions, yet there was no significant difference between nonclinician and clinician guidance. Despite the scarcity of longer follow-up data, it also appears that the positive effects of nonclinician-guided interventions persist beyond the intervention period. Taken together, our findings suggest that the use of nonclinicians is a promising way of incorporating cost-effective guidance into digital mental health interventions; their involvement can improve mental health outcomes to a degree on par with that achieved by professional mental health guidance. Interventions with guidance have improved outcomes compared to those without guidance, and have lasting effects.

There is often an assumption that clinical intervention requires highly trained professionals to optimize outcomes, despite research suggesting that the presence of human support alone increases adherence to digital mental health interventions, thereby yielding improved efficacy and outcomes [47]. In line with our findings, the presence of a guide—clinician or nonclinician—is beneficial for evoking positive changes. These results align with a review by Baumeister et al [13] that reported that changes in symptom severity did not differ significantly in groups supported by guides with differing levels of qualifications (n=4). Although Baumeister et al [13] considered clinical psychology students and psychologists without specialized postgraduate training as guides with lower qualifications, our study limited the designation of lower-qualified (nonclinician) guides to true nonclinicians, meaning graduate students in a mental health field were excluded. Still, our results indicate that Baumeister’s [13] findings (ie, that levels of effectiveness were comparable across levels of guide qualification) remain true with “lay” guides as well. This is particularly pertinent considering the push to increase the accessibility of mental health interventions (ie, through digital mental health platforms), given that these tools are likely to be more beneficial when supported by a guide. These results, therefore, show the possibility that larger-scale digital mental health interventions supported by personnel with lower levels of qualifications are feasible.

#### Adherence and Other Process Outcomes

With respect to adherence outcomes, the meta-analysis of the 3 studies we were able to pool showed no significant differences, although there appeared to be a trend toward higher adherence in the nonclinician-guided group relative to the unguided groups. The adherence results excluded in the meta-analysis were consistent with this trend. This is relatively unsurprising, given research suggesting that human support increases adherence by providing accountability [48]. However, there are limited studies reporting this metric, so additional information is needed.

With respect to other process outcomes, participants in all 6 studies that evaluated satisfaction reported high satisfaction across unguided, nonclinician-guided, and clinician-guided groups, though it is difficult to draw conclusions, as only 3 studies reported satisfaction in multiple groups. Furthermore, satisfaction measures included a heterogeneous landscape of satisfaction and usability scales, with many generated only for a specific study, and were prone to selection bias, as participants who are less satisfied with an intervention are more likely to drop out of the study. A more systematic understanding of how users perceive digital mental health interventions and which measures affect adherence would be gained if more studies reported standardized scales for process outcomes.

### Limitations of the Literature and Future Directions

Some included studies lacked a robust description of the roles and qualifications of the nonclinician guides. Within and across studies, nonclinician guides may have received a wide range of training and undertaken a variety of roles. Therefore, overall conclusions will not capture the likely heterogeneous effects of varying types of nonclinician support. Notably, 1 paper [26] included guides who utilized a psychosocial support approach through divulging anecdotes and reflecting upon their own recovery story to personally connect with participants. All other studies included in our analyses appeared to employ a supportive accountability model in which guides established participant accountability by creating, revising, and monitoring adherence goals and progress [48]. The inconsistencies and lack of detailed descriptions of the tasks performed by guides challenged our evaluation of which nonclinician roles were most effective, but this limitation likely reflects the infancy of this line of research. As nonclinician guidance appears beneficial in this context, future examination of the support type and the nonclinician guides’ training will be especially valuable in understanding how best to offer support that is effective and feasible within the digital mental health intervention format.

A similar issue (heterogeneity in definitions and measures) hampers the evaluation of adherence and other process outcomes. Intervention adherence differs from study attrition or dropout, as it refers to intervention uptake rather than study completion (eg, follow up). Study completion rates may not reflect the actual use of the intervention (eg, Christensen H et al [49]). The focus on adherence to digital interventions is related to whether the user engages with the tool, rather than whether they complete a follow-up assessment; this can be related more to study incentives than to intervention uptake. For this analysis, to minimize the risk of conflating intervention adherence with study completion, we defined adherence as the percentage of participants that completed all modules of the intervention, as it was the most-reported measure across the included studies. Two studies reported the percentage of participants who completed a “minimum dose” defined by the authors, and it has been posited that defining such minimum intended use may improve our understanding of intervention adherence [50]. Future studies should provide measures of intervention adherence that can be readily understood and differentiated from other variables, such as study completion.

### Limitations of the Current Study

Our results should be interpreted with caution due to several limitations. First, our search identified only 13 studies that assessed the effects of nonclinician guidance in digital mental health interventions through RCTs. Though we attempted to minimize the risk of missing studies by using a wide range of terms, we may have missed relevant studies given the lack of consensus around the terminology of this emerging category of nonclinician support. Further, our search was limited to English-language studies, which may have excluded studies that would have otherwise qualified for inclusion.

Another limitation was the inconsistency in methodology and the poor quality of many studies, which may hamper interpretation of results. Most studies were flagged as having “some concerns,” which aligns with the findings of other digital mental health systematic reviews [4,51]. To mitigate this limitation, we provided a structured assessment of bias as a general picture of the quality of the included studies. The duration between posttreatment and follow-up assessments also varied, and a wide range of sample sizes was found in our search. Notably, some papers included upwards of 100 participants in each trial arm [18,19,24], while other papers included approximately 30 participants in each trial arm. We evaluated our meta-analytic results to ensure study size did not unduly influence or skew the overall findings, but future evaluations of digital mental health interventions should aim to include more participants, in addition to standardizing follow-up assessments, to accurately capture lasting effects of the intervention.

Finally, while there was wide variation in heterogeneity across meta-analyses, the nonclinician versus control meta-analyses at both posttreatment and follow-up time points had moderate to high heterogeneity values (*I*^2^=75% and 58%, respectively) [52]. It appears the variation in control groups contributed to the heterogeneity; the type of control implemented by the study accounted for a large portion (94%) of heterogeneity. It is also important to note that we did not evaluate all possible explanatory variables through meta-regression, given our small number of eligible studies. Future meta-regressions should evaluate the effects of other factors, such as setting and population. Despite these limitations, our study provides valuable results in terms of next steps for this field of research, as well as allowing for a promising preliminary assessment of nonclinician guidance of digital interventions.

### Conclusion

Digital mental health interventions have emerged as a promising means of providing more accessible mental health care. This review demonstrates that nonclinician guidance yields more improvement in effectiveness outcomes than unguided or control interventions, and that nonclinician guidance can generate effectiveness outcomes comparable to those of clinician guidance in the context of digital mental health interventions. These results are encouraging, as integrating nonclinician guidance can increase the scalability and cost efficiency of digital interventions to meet the current demand for support. In particular, nonclinicians such as peers or technicians are much more readily available than clinicians and may be perceived as more relatable (eg, through having lived experience with mental health difficulties) and approachable (eg, it may be less stigmatizing to talk with a peer than a professional) by individuals seeking support. Incorporating nonclinician guides may be an advantageous way in which to facilitate access to effective support, since health system administrators and funding agencies may be more responsive to interventions that are likely to optimize benefits (ie, improved individual and community health and reduction in use of other services) but require relatively minimal resource demands. Further studies investigating the effects of guide qualification on digital health intervention effectiveness and process outcomes are needed and should clearly describe the specific roles of the guides, compare different levels of nonclinician support (eg, technician guidance vs psychosocial support), investigate the contributing mechanisms, and examine implementation feasibility for different types of guides.

## Appendix

Multimedia Appendix 1 Medline search strategy.

Multimedia Appendix 2 Summary of accepted papers.

Multimedia Appendix 3 Meta-analysis R code.

Multimedia Appendix 4 Cochrane Risk of Bias 2 Graphical Representation.

## Abbreviations

RCT: randomized controlled trial
RoB 2: Cochrane risk-of-bias tool version 2
SMD: standardized mean difference
