# Supplementary material for: The Effects of Nonclinician Guidance on Effectiveness and Process Outcomes in Digital Mental Health Interventions: Systematic Review and Meta-analysis
Source: J Med Internet Res. 2022 Jun 15;24(6):e36004. doi: 10.2196/36004 (PMC9244656; doi:10.2196/36004)
Supplement: Multimedia Appendix 1 [file jmir_v24i6e36004_app1.docx]

# Multimedia Appendix 1

Medline Search Strategy:

(internet-based intervention[MeSH] OR therapy, computer-assisted[MeSH] OR distance counseling[MeSH] OR (((intervention OR therapy OR treatment OR health) adj3 (internet* OR online OR web* OR electronic OR virtual OR computer* OR mobile OR app)) OR mobile health OR m-health OR mhealth OR e-health OR ehealth OR etool OR e-tool OR digital OR email OR e-mail OR text* OR messag* OR asynchron* OR synchron* OR distance counsel* OR e-counsel*)[mp] OR ((mobile OR phone) adj3 (app OR application))[mp]) AND

(exp mental disorders[MeSH] OR mental health[MeSH] OR exp stress, psychological[MeSH] OR exp substance-related disorders[MeSH] OR ((disorder* adj2 (behavior OR behaviour OR mental OR psychiatric OR bipolar OR eating OR mood OR personality OR psychotic OR panic OR obsessive compulsive OR obsessive-compulsive)) OR depression OR anxiety OR post-traumatic stress disorder OR post traumatic stress disorder OR PTSD OR schizophrenia)[mp] OR (mental health* OR (psychiatric adj2 (diagnosis OR disease* OR illness*)) OR stress* OR burnout OR subclinical OR subthreshold)[mp] OR (((abuse* OR addict* OR dependance OR habituation OR disorder* OR overdose* OR us*) adj2 (drug OR substance OR alcohol OR amphetamine OR cocaine OR inhalant OR marijuana OR thc OR cannab* OR hashish OR narcotic OR opioid* OR tobacco OR nicotine OR hallucinogen* OR sedative* OR meth* OR heroin OR MDMA OR stimulant)) OR alcoholi*)[mp]) AND

(mental health services[MeSH] OR community mental health services[MeSH] OR counseling[MeSH] OR directive counseling[MeSH] OR social support[MeSH] OR exp psychotherapy[MeSH] OR distance counseling[MeSH] OR (psychotherap* OR therap*)[mp] OR (mental health service* OR counseling OR counselling OR psychological support OR psychoeducation OR support)[mp] OR (distance counseling OR distance counselling)[mp]) AND

((community health workers[MeSH] OR mentoring[MeSH] OR (((community OR lay) adj2 (worker* OR aide* OR personnel OR provider)) OR task-shift* OR taskshift* OR task shift* OR taskshar* OR task shar* OR task-shar* OR administrative engagement OR lay worker* OR lived experience OR peer* OR caregiver* OR technician* OR research assistant*OR health auxiliar*)[mp]) OR ((guidance OR guide* OR e-coach* OR ecoach* OR e coach*)[mp] NOT (psychiatrist* OR psychologist* OR clinician* OR nurse* OR specialist* OR counselor* OR counsellor* OR therapist* OR social worker*)[mp])) AND

(adult[MeSH] OR adolescent[MeSH] OR young adult[MeSH] OR middle aged[MeSH] OR (adult* or youth or adolescent* or student* or college or university or young person or young people)[mp]) AND

(exp clinical trial[MeSH] OR evaluation study[MeSH] OR (((clinical OR control* OR clinical OR randomiz*) adj2 trial*) OR evaluation stud* OR RCT* OR quasi?experiment*)[mp])

limit to (english language and last 10 years)
