# Supplementary material for: The Effects of Nonclinician Guidance on Effectiveness and Process Outcomes in Digital Mental Health Interventions: Systematic Review and Meta-analysis
Source: J Med Internet Res. 2022 Jun 15;24(6):e36004. doi: 10.2196/36004 (PMC9244656; doi:10.2196/36004)
Supplement: Multimedia Appendix 2 [file jmir_v24i6e36004_app2.docx]

# Multimedia Appendix 2

| Study | Location | Targeted Disorder | Conditions | Duration | Non-clinician Guide Qualification | Theoretical Model |
| --- | --- | --- | --- | --- | --- | --- |
| An et al., 2013 | | | | | | |
|  | USA | Substance use (smoking) | 1. General interest lifestyle content (n=476) 2. Tailored health messages   (n=473)   1. Tailored health messages with peer coaching   (n=456) | 6 week duration, 7 week post-enrolment assessment, 12 week follow-up | Undergraduates or recent graduates with 3-day motivational interviewing training | Social Cognitive Theory, Theory of Reasoned Action and Planned Behaviour, Self-determination Theory |
| Arjadi et al., 2018 | | | | | | |
|  | Indonesia | Depression | 1. Guided Act and Feel Indonesia (n=159) 2. Online psychoeducation (n=154) | 8 week duration, 10 week post-enrolment assessment, 3 and 6 month follow-up | Lay support counsellors with 2 day program training | Behavioural Activation |
| Day et al., 2013 | | | | | | |
|  | Canada | Depression  Anxiety  Stress | 1. CBT program (n=33) 2. Delayed access control (n=33) | 6 week duration, 6 week post-enrolment assessment, 6 month post-enrolment follow-up | Trained undergraduate/graduate students | CBT |
| Dirkse et al., 2020 | | | | | | |
|  | Canada | Depression, Anxiety | 1. Technician guided iCBT (N = 41) 2. Self-guided iCBT (N = 42) | 8 week duration, 8 week post-enrolment assessment, 1 month follow-up | Technician | CBT |
| Farrer et al., 2011 | | | | | | |
|  | Australia | Depression | 1. Program only (N = 38) 2. Program with phone tracking (N = 41) 3. Phone Tracking only (N = 39) 4. Control (N = 35) | 6 week duration, 6 week post-enrolment assessment, 8 month intervention follow-up | Lay telephone counsellor | Online psychoeducation with CBT |
| Flynn et al., 2020 | | | | | | |
|  | United Kingdom | Mental Well-being | 1. Be Mindful (N = 30) 2. Be Mindful with Peer Mentoring (N = 30) | Minimum 4 weeks duration, 12 week and 6 month post-enrolment follow-up | Parent carers of people with intellectual disabilities with 1 day of program training | Mindfulness Based Cognitive Therapy (MBCT) |
| Heber et al., 2016 | | | | | | |
|  | Germany | Stress | 1. Internet-based stress management intervention (N = 132) 2. Waitlist control (N = 132) | 7 week duration, 7 week post-intervention assessment, 6 and 12 month follow-up for intervention group only | e-Coaches with a degree in Psychology | Lazarus’ Transactional Model of Stress |
| Kobak et al., 2015 | | | | | | |
|  | USA | Obsessive Compulsive Disorder | 1. BT Steps with lay coaching (n=28) 2. BT Steps with therapist coaching (n=31) 3. BT Steps with no coaching (n=28) | 12 week duration, 12 week post-enrolment assessment | Lay coach | CBT |
| Possemato et al., 2019 | | | | | | |
|  | USA | PTSD and hazardous drinking | 1. Thinking Forward with peer support (n=15) 2. Thinking Forward (n=15) | 12 week duration, 12 week post-enrolment assessment, 24 week follow-up | Peer supporter with 13 hours of study-specific training over 10 weeks | - |
| Proudfoot et al., 2012 | | | | | | |
|  | Australia | Bipolar Disorder (perception of illness) | 1. Bipolar Education Program with peer support (n=139) 2. Bipolar Education Program (n=141) 3. Control – weekly informative emails (n=139) | 8 week duration, 8 week post-enrolment assessment, 3 and 6 month post-enrolment follow-up | Informed supporters with lived experience of BD | - |
| Robinson et al., 2010 | | | | | | |
|  | Australia | Generalized Anxiety Disorder | 1. Worry Program with technician support (n=50) 2. Worry program with clinician support (n=47) 3. Control – delayed access (n=48) | 10 week duration, 10 week post-enrolment assessment, 3 month follow-up | Technician (clinic manager) | CBT |
| Rosso et al., 2017 | | | | | | |
|  | USA | Depression | 1. iCBT with peer support (n=37) 2. Control – monitored attention control (n=40) | 10 week duration, 10 week post-enrolment assessment | Trained bachelor-level research assistant | CBT |
| Titov et al., 2010 | | | | | | |
|  | Australia | Depression | 1. Sadness program with technician support (n=41) 2. Sadness program with clinician support (n=46)   Control (n=40) | 8 week duration, 8 week post-enrolment assessment, 4 month post-enrolment follow-up | Technician (clinic manager) | CBT |
