# Supplementary material for: The Effects of Nonclinician Guidance on Effectiveness and Process Outcomes in Digital Mental Health Interventions: Systematic Review and Meta-analysis
Source: J Med Internet Res. 2022 Jun 15;24(6):e36004. doi: 10.2196/36004 (PMC9244656; doi:10.2196/36004)
Supplement: Multimedia Appendix 3 [file jmir_v24i6e36004_app3.docx]

# Multimedia Appendix 3

#Meta-analysis#

#### LIBRARIES ####

library(meta)

library(metafor)

library(esc)

#### Hedge's g Calculations ####

#T1 Non-clinician vs. Control

#grp1 = Non-clinician; grp2 = Control

#An et al. reverse coded for abstinence

esc_bin_prop(prop2event = 0.14, grp2n = 456,

prop1event = 0.09, grp1n = 476, es.type = "g")

#Arjadi et al.

esc_mean_sd(grp1m = 8.5, grp1sd = 5.74, grp1n = 120,

grp2m = 10.83, grp2sd = 6.21, grp2n = 145, es.type = "g")

#Day et al.

esc_mean_sd(grp1m = 10.43, grp1sd = 4.49, grp1n = 24,

grp2m = 14.6, grp2sd = 9.51, grp2n = 29, es.type = "g")

#Rosso et al.

esc_mean_sd(grp1m = 9.17, grp1sd = 6.92, grp1n = 30,

grp2m = 14.05, grp2sd = 5.34, grp2n = 30, es.type = "g")

#Heber et al.

esc_mean_sd(grp1m = 17.88, grp1sd = 6.17, grp1n = 116,

grp2m = 22.96, grp2sd = 6.07, grp2n = 127, es.type = "g")

#Robinson et al.

esc_mean_sd(grp1m = 6.02, grp1sd = 3.43, grp1n = 45,

grp2m = 11.25, grp2sd = 4.7, grp2n = 47, es.type = "g")

#Titov et al.

esc_mean_sd(grp1m = 15.29, grp1sd = 9.81, grp1n = 37,

grp2m = 26.15, grp2sd = 10.14, grp2n = 39, es.type = "g")

#Farrer et al.

esc_mean_sd(grp1m = 21, grp1sd = 12.4, grp1n = 18,

grp2m = 35.1, grp2sd = 13.9, grp2n = 27, es.type = "g")

#T1 Non-clinician vs. Unguided

#grp1 = Non-clinician; grp2 = Unguided

#An et al. reverse coded for abstinence

esc_bin_prop(prop2event = 0.14, grp2n = 456,

prop1event = 0.11, grp1n = 473, es.type = "g")

#Flynn et al. reverse coded - high WEMWBS score = better mental well-being

esc_mean_sd(grp2m = 46.43, grp2sd = 12.66, grp2n = 21,

grp1m = 42.88, grp1sd = 9.66, grp1n = 25, es.type = "g")

#Dirkse et al.

esc_mean_sd(grp1m = 4.83, grp1sd = 2.7, grp1n = 40,

grp2m = 5.51, grp2sd = 4.5, grp2n = 41, es.type = "g")

#Possemato et al.

esc_mean_sd(grp1m = 41.78, grp1sd = 14.9, grp1n = 9,

grp2m = 43.16, grp2sd = 13.42, grp2n = 11, es.type = "g")

#Kobak et al.

esc_mean_sd(grp1m = 15.61, grp1sd = 5.88, grp1n = 28,

grp2m = 16.32, grp2sd = 6.97, grp2n = 28, es.type = "g")

#Farrer et al.

esc_mean_sd(grp1m = 21, grp1sd = 12.4, grp1n = 18,

grp2m = 24.4, grp2sd = 13.6, grp2n = 27, es.type = "g")

#T1 Non-clinician vs. Clinician

#grp1 = Non-clinician; grp2 = Clinician

#Robinson et al.

esc_mean_sd(grp1m = 6.02, grp1sd = 3.43, grp1n = 45,

grp2m = 5.55, grp2sd = 4.73, grp2n = 46, es.type = "g")

#Titov et al.

esc_mean_sd(grp1m = 15.29, grp1sd = 9.81, grp1n = 37,

grp2m = 14.59, grp2sd = 11.12, grp2n = 41, es.type = "g")

#Kobak et al.

esc_mean_sd(grp1m = 15.61, grp1sd = 5.88, grp1n = 28,

grp2m = 15.32, grp2sd = 7.04, grp2n = 31, es.type = "g")

#T2 Non-clinician vs. Control

#grp1 = Non-clinician; grp2 = Control

#An et al. reverse coded for abstinence

esc_bin_prop(prop2event = 0.31, grp2n = 430,

prop1event = 0.11, grp1n = 446, es.type = "g")

#Heber et al.

esc_mean_sd(grp1m = 16.08, grp1sd = 6.03, grp1n = 115,

grp2m = 22.1, grp2sd = 5.81, grp2n = 121, es.type = "g")

#Farrer et al.

esc_mean_sd(grp1m = 18.4, grp1sd = 10.4, grp1n = 18,

grp2m = 34.2, grp2sd = 13.5, grp2n = 22, es.type = "g")

#T2 Non-clinician vs. Unguided

#grp1 = Non-clinician; grp2 = Unguided

#An et al. reverse coded for abstinence

esc_bin_prop(prop2event = 0.31, grp2n = 430,

prop1event = 0.23, grp1n = 441, es.type = "g")

#Flynn et al. reverse coded - high WEMWBS score = better mental well-being

esc_mean_sd(grp2m = 47.43, grp2sd = 10.69, grp2n = 23,

grp1m = 43.52, grp1sd = 10.23, grp1n = 27, es.type = "g")

#Dirkse et al.

esc_mean_sd(grp1m = 3.59, grp1sd = 2.4, grp1n = 39,

grp2m = 4.8, grp2sd = 3.2, grp2n = 38, es.type = "g")

#Possemato et al.

esc_mean_sd(grp1m = 40.31, grp1sd = 12.1, grp1n = 9,

grp2m = 41.64, grp2sd = 12.18, grp2n = 11, es.type = "g")

#Farrer et al.

esc_mean_sd(grp1m = 18.4, grp1sd = 10.4, grp1n = 18,

grp2m = 18, grp2sd = 13, grp2n = 23, es.type = "g")

#T2 Non-clinician vs. Clinician

#grp1 = Non-clinician; grp2 = Clinician

#Robinson et al.

esc_mean_sd(grp1m = 6.26, grp1sd = 3.64, grp1n = 38,

grp2m = 5.55, grp2sd = 5.14, grp2n = 33, es.type = "g")

#Titov et al.

esc_mean_sd(grp1m = 11.66, grp1sd = 9.58, grp1n = 30,

grp2m = 16.22, grp2sd = 13.16, grp2n = 38, es.type = "g")

#### Meta-analyses, Forest Plots, Funnel Plots ####

#T1 Non-clinician vs. Control

data1<-T1_Non_clinician_vs_Control

T1Nonclinician.Control<- metagen(TE,

seTE,

data = data1,

studlab = paste(Author),

comb.fixed = FALSE,

comb.random = TRUE,

method.tau = "SJ",

hakn = TRUE,

prediction = TRUE,

sm = "SMD")

forest.meta(T1Nonclinician.Control,

leftlabs = c("Author", "Hedges' g","Standard Error"),

text.random = "Overall effect",

comb.random = TRUE,

comb.fixed = FALSE,

print.tau2 = FALSE,

print.pval.Q = FALSE,

prediction = FALSE,)

#T1 Non-clinician vs. Unguided

data2<-T1_Non_clinician_vs_Unguided

T1Nonclinician.Unguided<- metagen(TE,

seTE,

data = data2,

studlab = paste(Author),

comb.fixed = FALSE,

comb.random = TRUE,

method.tau = "SJ",

hakn = TRUE,

prediction = TRUE,

sm = "SMD")

forest.meta(T1Nonclinician.Unguided,

leftlabs = c("Author", "Hedges' g","Standard Error"),

text.random = "Overall effect",

comb.random = TRUE,

comb.fixed = FALSE,

print.tau2 = FALSE,

print.pval.Q = FALSE,

prediction = FALSE,)

#T1 Non-clinician vs. Clinician

data3<-T1_Non_clinician_vs_Clinician

T1Nonclinician.Clinician<- metagen(TE,

seTE,

data = data3,

studlab = paste(Author),

comb.fixed = FALSE,

comb.random = TRUE,

method.tau = "SJ",

hakn = TRUE,

prediction = TRUE,

sm = "SMD")

forest.meta(T1Nonclinician.Clinician,

leftlabs = c("Author", "Hedges' g","Standard Error"),

text.random = "Overall effect",

comb.random = TRUE,

comb.fixed = FALSE,

print.tau2 = FALSE,

print.pval.Q = FALSE,

prediction = FALSE,)

#T2 Non-clinician vs. Control

data4<-T2_Non_clinician_vs_Control

T2Nonclinician.Control<- metagen(TE,

seTE,

data = data4,

studlab = paste(Author),

comb.fixed = FALSE,

comb.random = TRUE,

method.tau = "SJ",

hakn = TRUE,

prediction = TRUE,

sm = "SMD")

forest.meta(T2Nonclinician.Control,

leftlabs = c("Author", "Hedges' g","Standard Error"),

text.random = "Overall effect",

comb.random = TRUE,

comb.fixed = FALSE,

print.tau2 = FALSE,

print.pval.Q = FALSE,

prediction = FALSE,)

#T2 Non-clinician vs. Unguided

data5<-T2_Non_clinician_vs_Unguided

T2Nonclinician.Unguided<- metagen(TE,

seTE,

data = data5,

studlab = paste(Author),

comb.fixed = FALSE,

comb.random = TRUE,

method.tau = "SJ",

hakn = TRUE,

prediction = TRUE,

sm = "SMD")

forest(T2Nonclinician.Unguided,

leftlabs = c("Author", "Hedges' g","Standard Error"),

text.random = "Overall effect",

comb.random = TRUE,

comb.fixed = FALSE,

print.tau2 = FALSE,

print.pval.Q = FALSE,

prediction = FALSE,)

#Adherence Meta-analysis

flynn<-escalc(measure = "OR",ai = 14, bi = 13, n1i = 27, ci = 13, di = 17, n2i = 30)

summary(flynn)

dirkse<-escalc(measure = "OR", ai = 38, bi = 3, ci = 34, di = 8)

summary(dirkse)

farrer<-escalc(measure = "OR", ai = 8, bi = 37, ci = 6, di = 32)

summary(farrer)

rdadherence<-oddsratio_adherence

rdadherencemeta<-metagen(TE,

seTE,

studlab = paste(Author),

data = rdadherence,

comb.fixed = FALSE,

comb.random = TRUE,

method.tau = "SJ",

hakn = TRUE,

prediction = TRUE,

sm = "OR")

forest(rdadherencemeta,

leftlabs = c("Author", "OR","Standard Error"),

text.random = "Overall effect",

comb.random = TRUE,

comb.fixed = FALSE,

print.tau2 = FALSE,

print.pval.Q = FALSE,

prediction = FALSE,)

#### Meta-Regression ####

# Non-clinician vs. Control, Post-Treatment - Control Group #

#An - alternative intervention

#Arjadi - alternative intervention

#Day - no intervention

#Farrer - no intervention

#Heber - no intervention

#Robinson - no intervention

#Rosso - alternative intervention

#Titov - no intervention

#no intervention = 0; alternative intervention = 1

control<- c(1, 1, 0, 0, 0, 0, 1, 0)

T1metaregcontrol<- metareg(T1Nonclinician.Control, ~control, method.tau = "REML")

bubble(T1metaregcontrol, studlab = TRUE)

# Non-clinician vs. Control, Follow-up #

#An - alternative intervention

#Farrer - no intervention

#Heber - no intervention

controlt2<- c(1, 0, 0)

T2metaregcontrol <- metareg(T2Nonclinician.Control, ~controlt2, method.tau = "REML")
