# Supplementary figures and images for: The Effects of Nonclinician Guidance on Effectiveness and Process Outcomes in Digital Mental Health Interventions: Systematic Review and Meta-analysis
Source: J Med Internet Res. 2022 Jun 15;24(6):e36004. doi: 10.2196/36004 (PMC9244656; doi:10.2196/36004)

# Multimedia Appendix 4

Graphical Representations of Cochrane RoB 2 Assessments


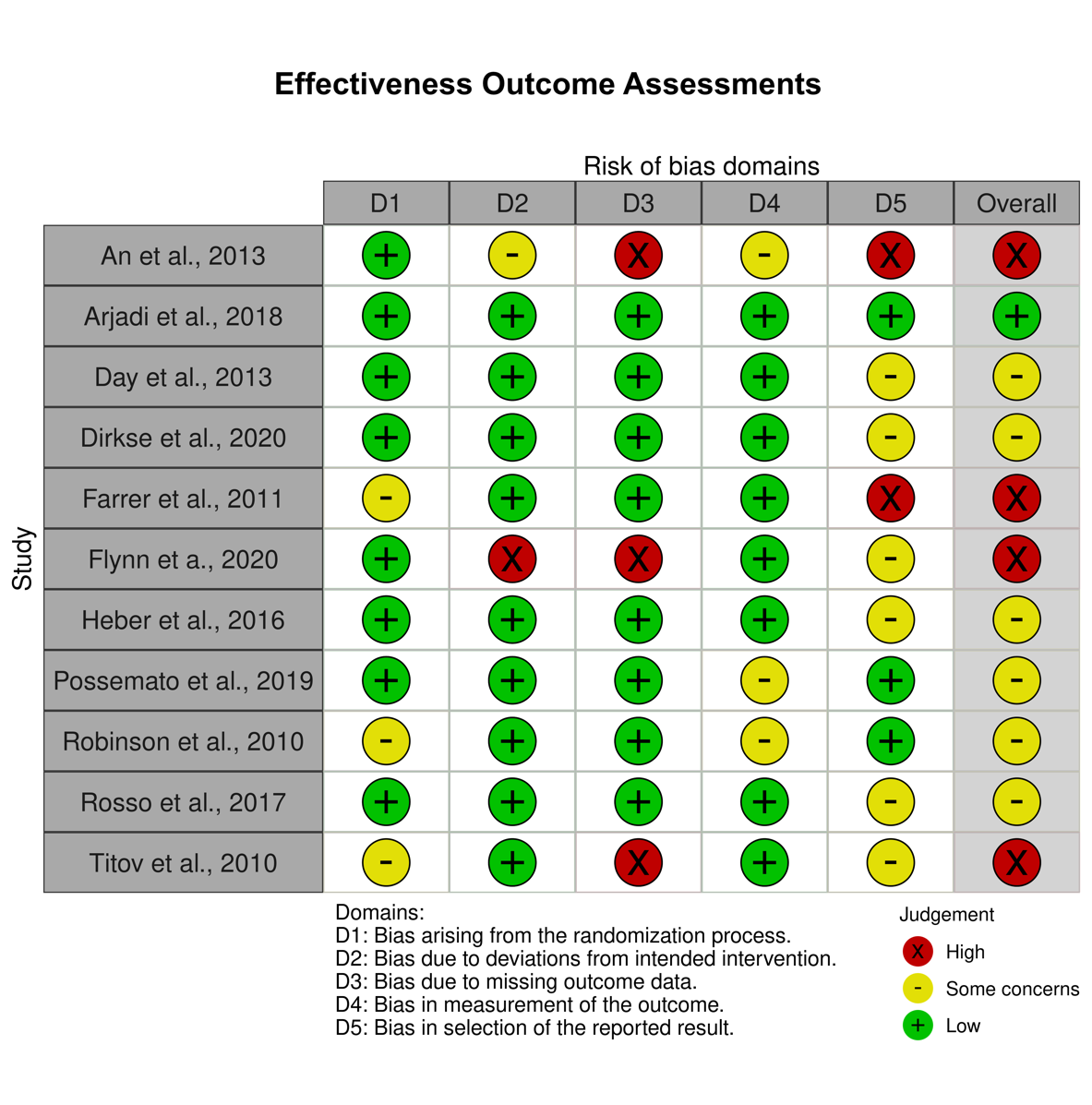


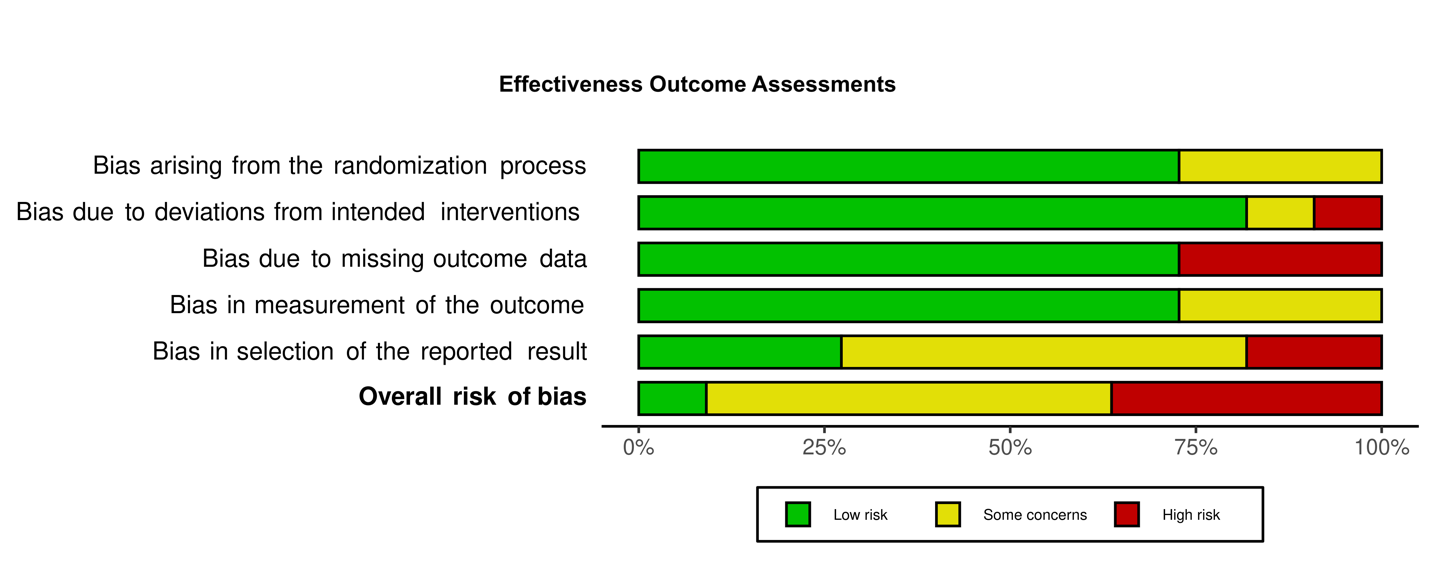


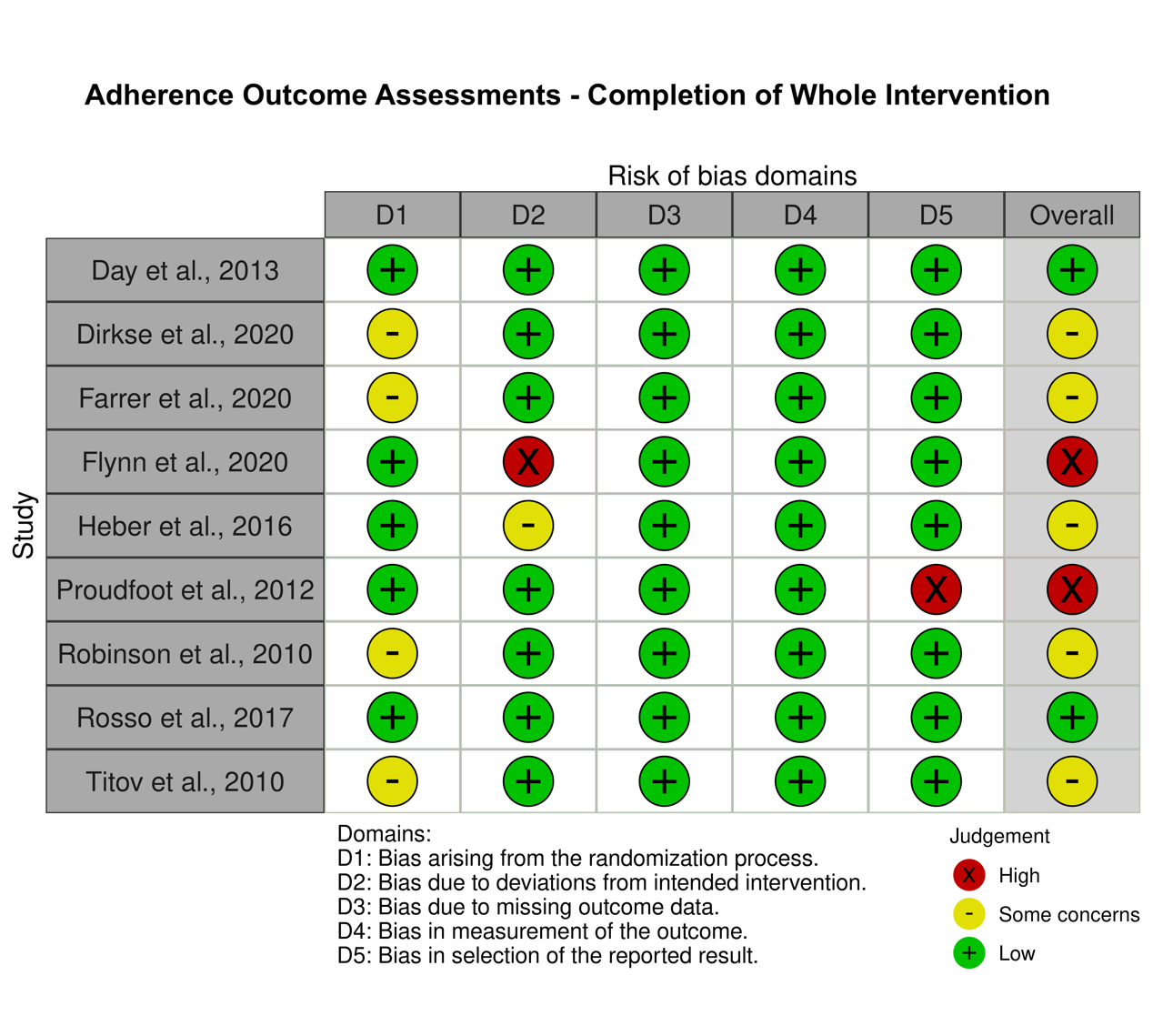

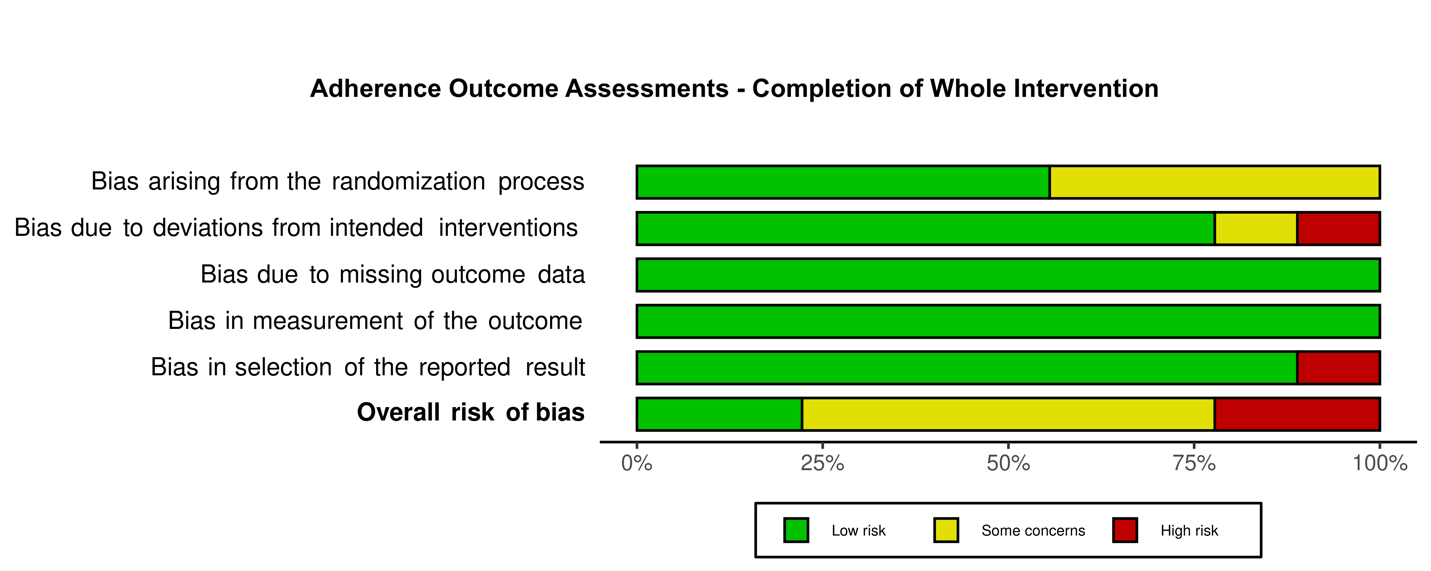


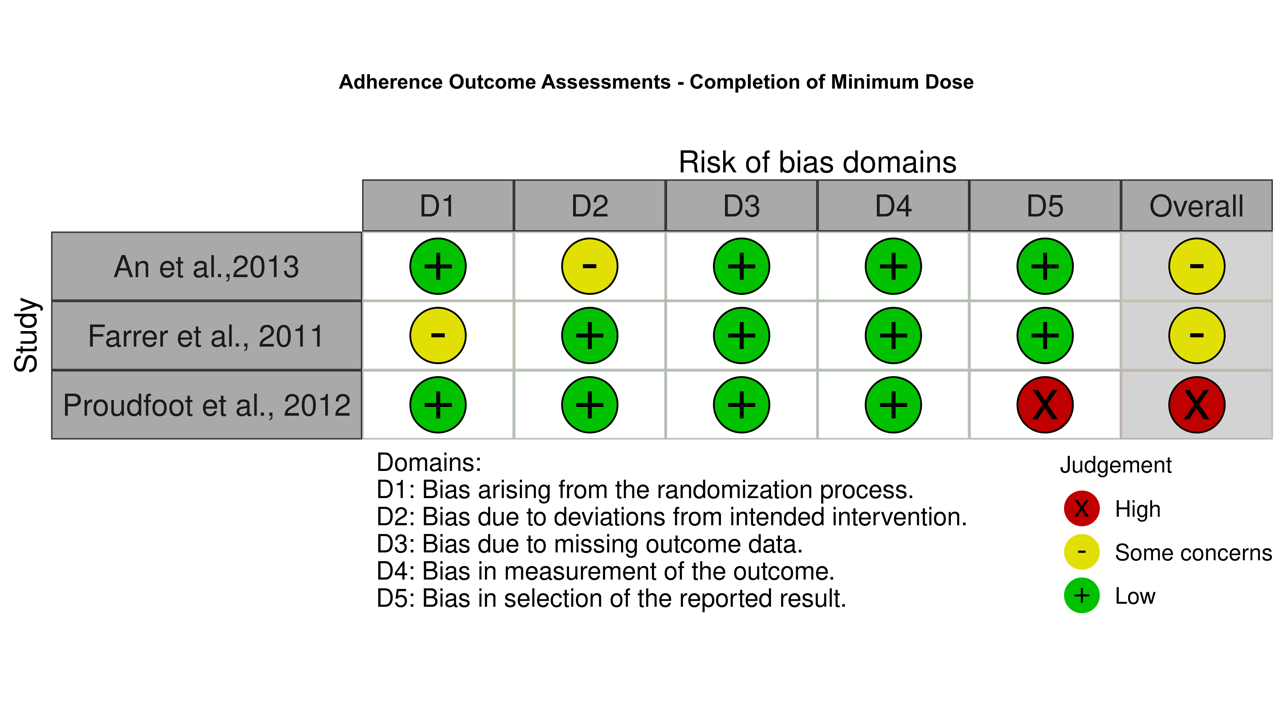

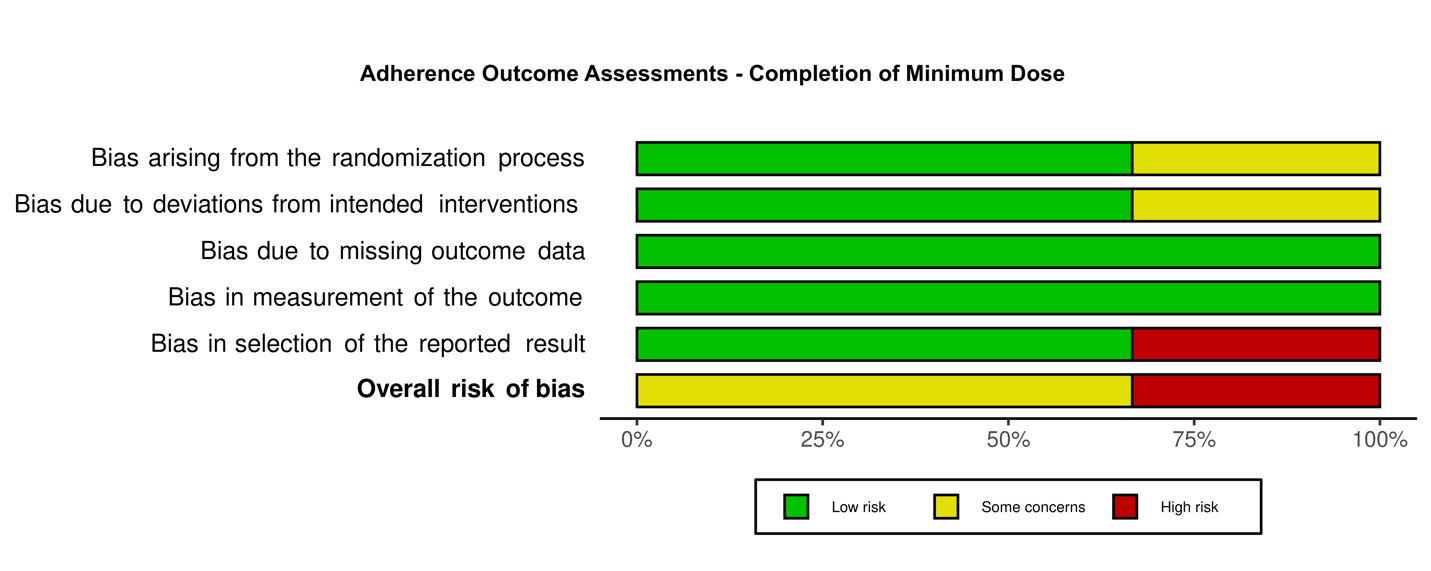


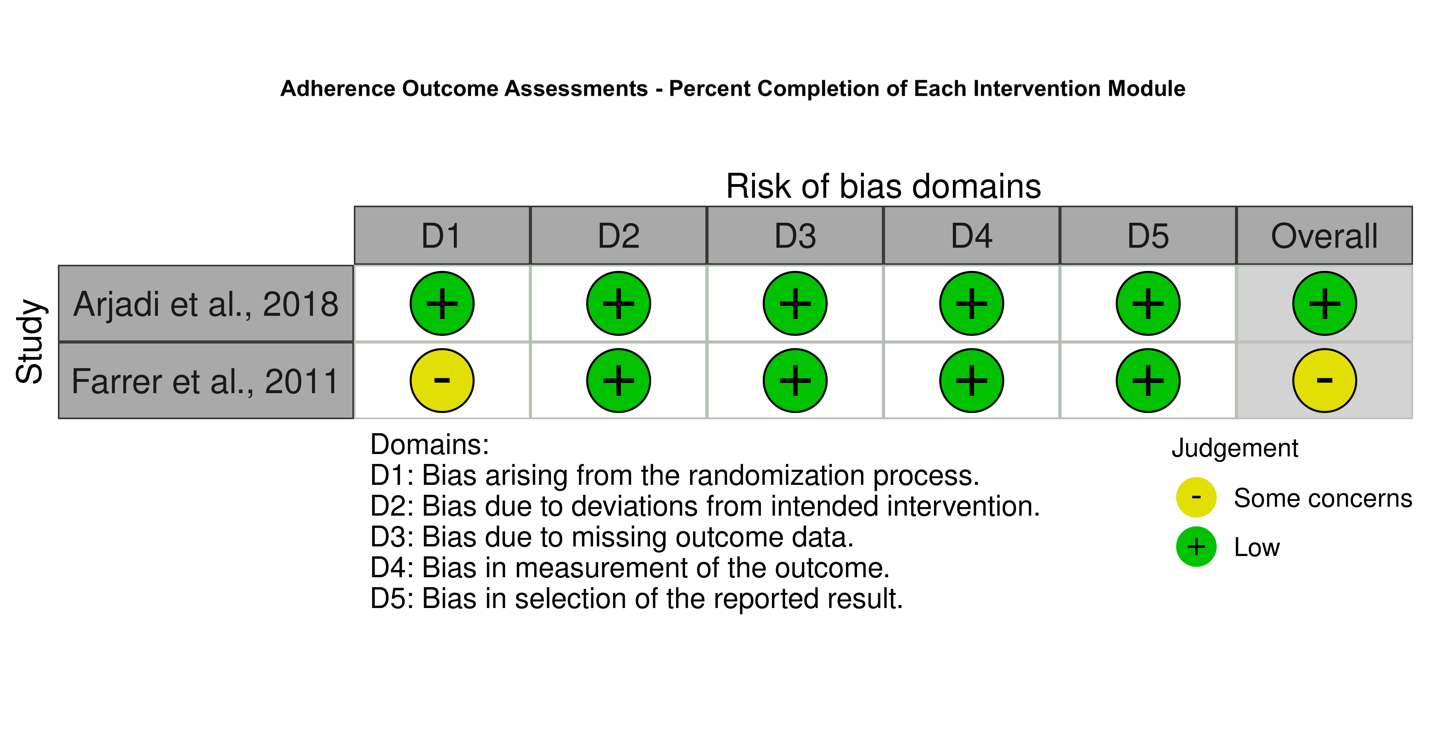

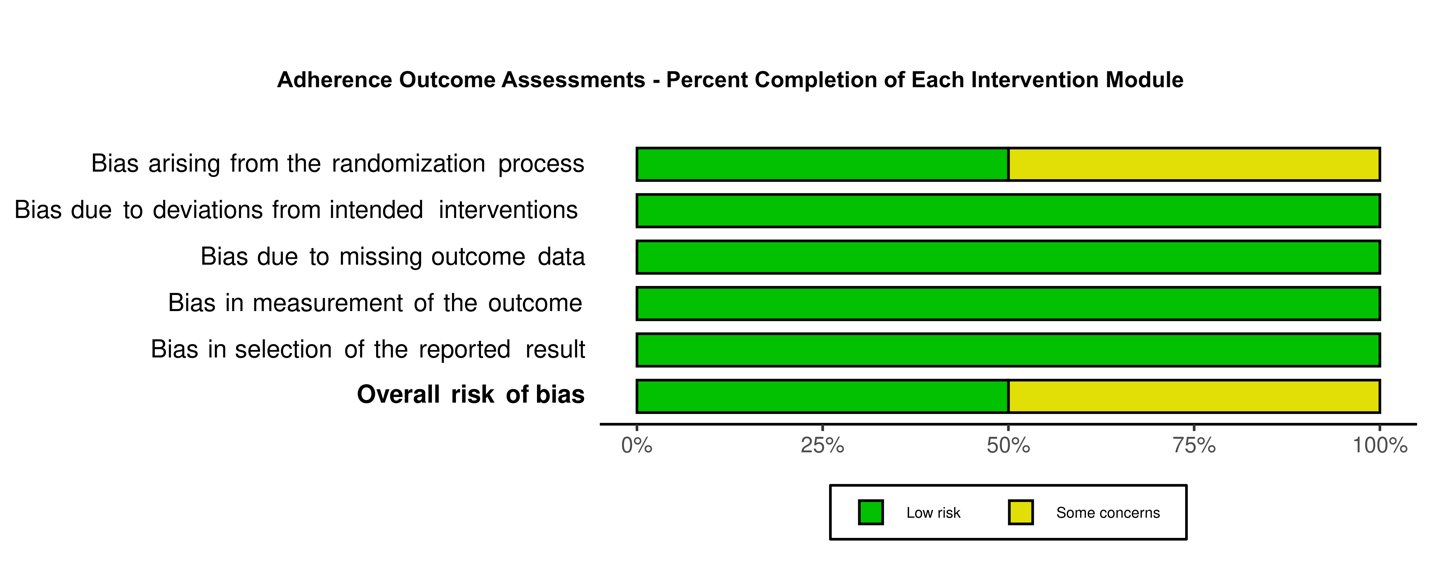


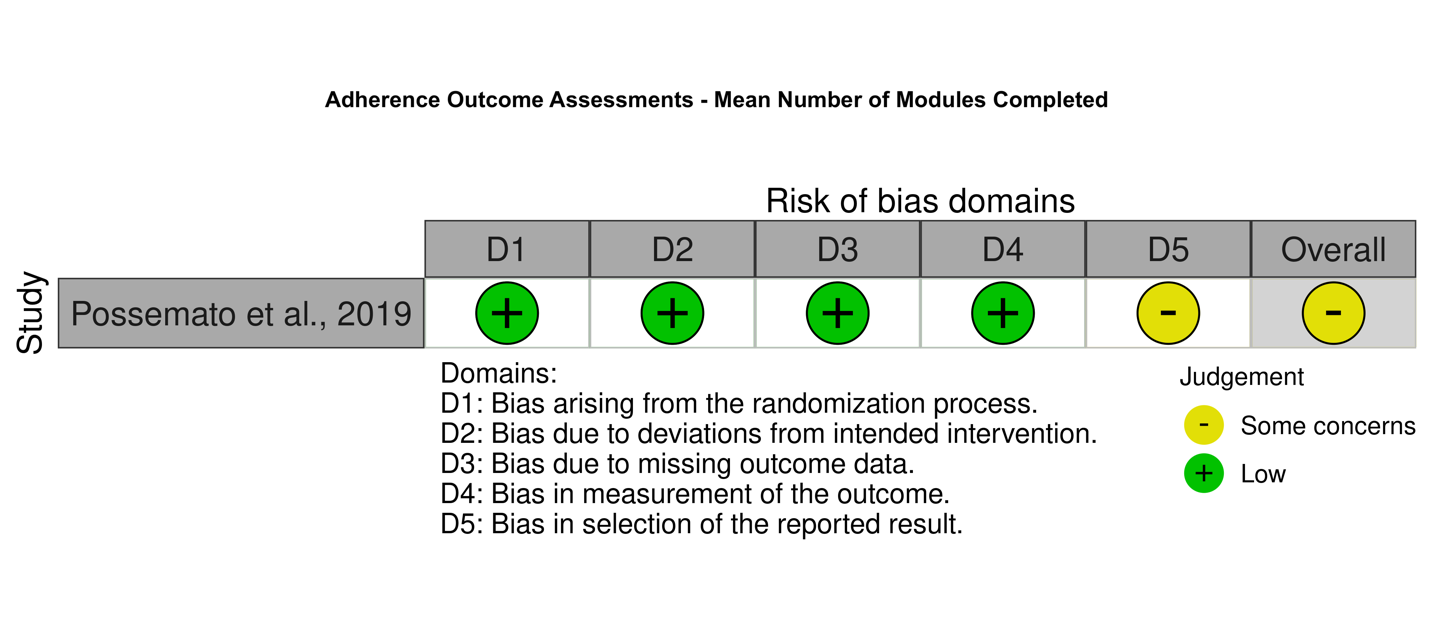

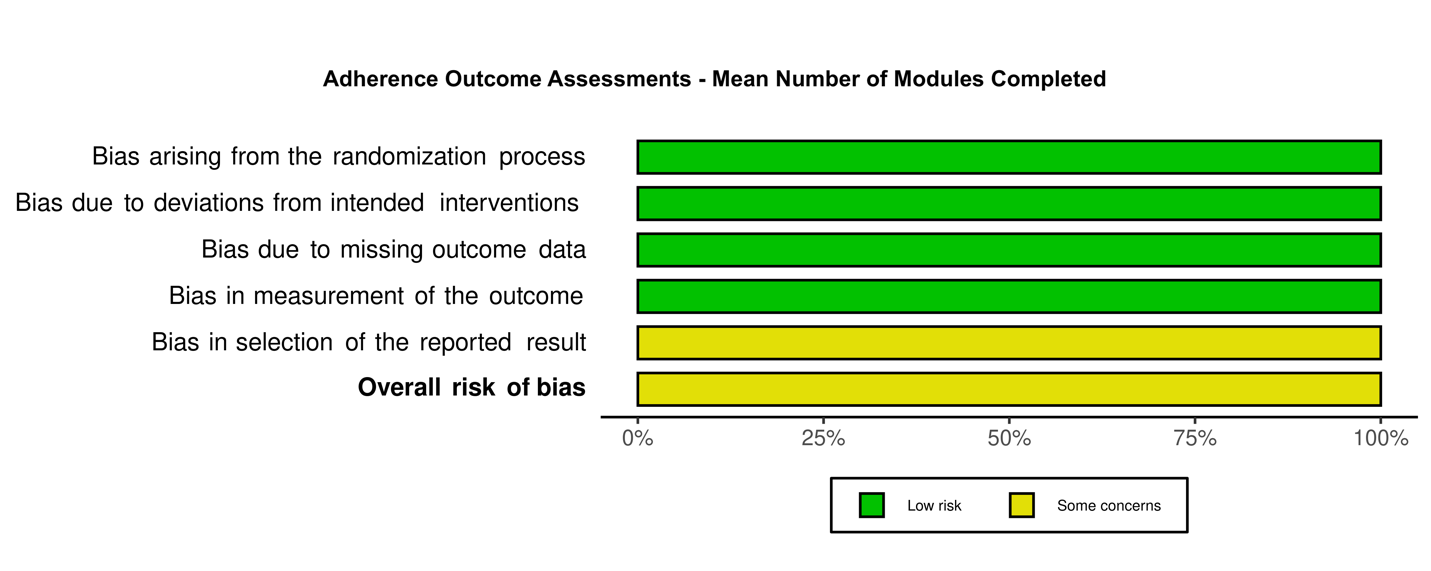


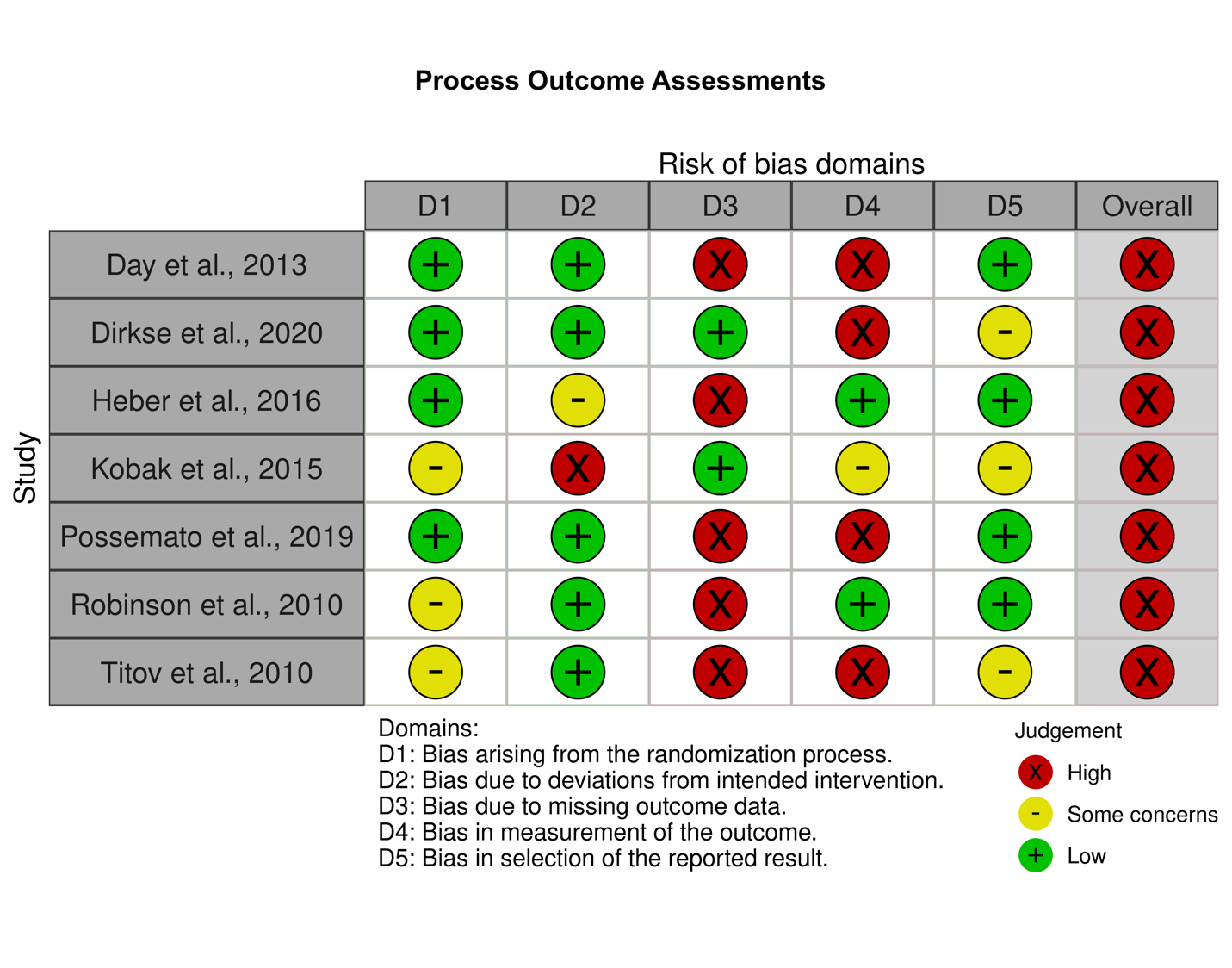

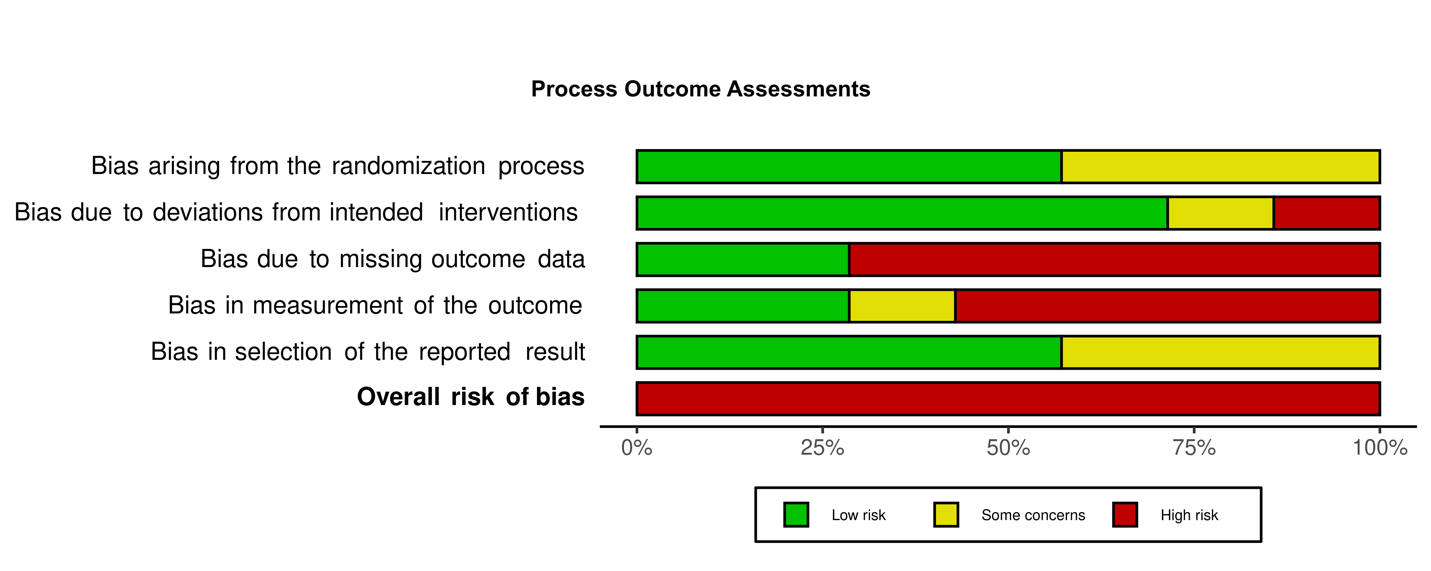

Supplement: Multimedia Appendix 4 [file jmir_v24i6e36004_app4.docx]
